# The association between serum selenium concentration and prognosis in patients with heart failure in a Chinese population

**DOI:** 10.1038/s41598-021-93873-7

**Published:** 2021-07-15

**Authors:** Zhiliang Zhang, Chao Chang, Yuxin Zhang, Zhiyong Chai, Jinbei Li, Chunguang Qiu

**Affiliations:** 1 Department of Cardiology, The First Affiliated Hospital of Zhengzhou University, No. 1 Jianshe East Road, Zhengzhou, 450052 Henan China; 2 Department of Cardiology, Nanyang Central Hospital, NanYang, 473000 Henan China; 3 Department of Cardiology, The Second Affiliated Hospital of Zhengzhou University, Zhengzhou, 450014 Henan China

**Keywords:** Cardiology, Heart failure

## Abstract

Whether Selenium (Se) deficiency relates with adverse prognosis in Chinese patients with heart failure (HF) is still unknown. This study aimed to investigate the association of serum Se level and the outcomes of patients with HF in a Chinese population. Patients with HF and serum Se examination were retrospectively included. Baseline information were collected at patient’s first admission. The primary and secondary outcomes were all-cause mortality and rehospitalization for HF during follow-up, respectively. The study participants were divided into quartiles according to their serum Se concentrations. The Cox proportional hazard models were adopted to estimate the association of serum Se levels with observed outcomes. A total of 411 patients with HF with a mean age of 62.5 years were included. The mean serum level of Se was 68.3 ± 27.7 µg/L. There was nonsignificant difference of baseline characterizes between the four quartile groups. In comparison with patients in the highest quartile, those with the lowest quartile (17.40–44.35 µg/L) were associated with increased risk of all-cause mortality [adjusted hazard ratios (95% CI) 2.32 (1.43–3.77); *P*_trend_ = 0.001]. Our study suggested that a lower serum Se level was significantly associated with increased risk of all-cause mortality in patients with HF.

## Introduction

Heart failure (HF) is an important disease burden worldwide with a 1–2% prevalence among global population^1^. Despite numerous advances in therapeutics of HF, there are still unmet needs to improve quality of life and long-term survival of those patients^2^. Patients with HF, especially for elderly, are commonly presented with malnutrition and micronutrients deficiency as underdetermined reasons^3,4^. Over last decade, several studies have found the associations between some trace elements deficiency (e.g. Zn, Fe) and adverse clinical outcomes in patients with HF^5,6^. Moreover, iron supplement has been evidenced to benefit HF patients with iron deprivation regarding to symptoms and exercise capacity^7,8^.

Selenium (Se) is an essential trace element for human health. Selenocysteine, the active form of Se, is incorporated as 25 selenoproteins including glutathione peroxidase (GPx), thioredoxin reductase, selenoprotein-P, redox-regulating signaling, and thyroid hormones metabolism^9^. The soil content of selenium is varying greatly, which can affect dietary selenium intake. For example, Venezuela, Canada, the United States and Japan have high intakes of Se (> 100 μg/day)^10^. In contrast, China has areas both with selenium deficiency and excess.

Severe Se deficiency has been proofed as a cause of congestive HF, a disorder known as Keshan Disease^11^, and less severe Se deficiency or suboptimal Se status has also been suggested associated with HF progression^12^. Recently, Bomer et al. using an European cohort of 2516 patients with HF reported that approximately 25% of patients with worsening HF have a serum Se < 70 µg/L, and that was associated with a poor quality of life and poor exercise capacity, as well as a worse prognosis^13^. However, serum Se levels vary greatly within different populations. In some regions such as China, Se deficiency maybe more common because of the Se-deficient soil and diet^10,14^. The prevalence of Se deficiency and whether it relates with adverse prognosis in Chinese patients with HF were still unknown.

Therefore, this study aimed to investigate the association of serum Se level and the outcomes of patients with HF in a Chinese population.

## Results

### Patient selection and baseline characteristics

Between January 2015 and December 2018, 635 patients with confirmed HF were initial screened. Among them, 113 patients were excluded due to the absence of serum Se measurement. Sixty-seven patients were further excluded by thyroid disease (n = 42), treatment with amiodarone or glucocorticoids (n = 54), and severe systemic disease (n = 25). A total of 411 patients with HF with a mean age of 62.5 years were finally included (Table 1). The patients were less often female (44.5%), and had a markedly elevated level of N-terminal pro-B type natriuretic peptide (NT-proBNP) (3541 [IQR 1952–6909] pg/mL). The 80% of patients were in New York Heart Association (NYHA) function class III-IV. A majority of patients (71.8%) were identified as having a prior history of HF, the mean duration of HF was 20 ± 14 months. Nearly one-fourth of patients presented with acute or worsening decompensated HF at admission. According to left ventricle ejection fraction (EF), the prevalence was 47.2% of HF with preserved EF (n = 194), as well 20.4% (n = 84) of HF with mid-range EF and 32.4% (n = 132) of HF with reduced EF.

**Table 1:** Baseline characteristics of the study population stratified by quartile of serum Se concentration.

Variables	Total	1st quartile (17.4–44.35 µg/L) (n = 104)	2nd quartile (44.35–68.05 µg/L) (n = 102)	3rd quartile (68.05–94.15 µg/L) (n = 103)	4th quartile (94.15–116.7 µg/L) (n = 102)	*P*
Age (years)	62.5 ± 15.9	60.0 ± 17.0	62.1 ± 16.5	63.8 ± 15.8	64.0 ± 14.0	0.223
Female	183 (44.5%)	45 (43.3%)	48 (47.1%)	47 (45.6%)	43 (42.2%)	0.893
BMI (kg/m^2^)	26.7 ± 4.7	27.6 ± 4.6	26.4 ± 4.5	25.8 ± 4.8	26.9 ± 4.7	0.596
Current smoker	82 (20.0%)	18 (17.3%)	19 (18.6%)	16 (15.5%)	29 (28.4%)	0.093
Current drinker	61 (14.8%)	15 (14.4%)	17 (16.8%)	9 (8.7%)	20 (19.6%)	0.562
**Clinical features**						
Prior history of HF	295 (71.8%)	80 (76.9%)	69 (67.6%)	71 (68.9%)	75 (73.5%)	0.426
HF duration (months)	20.4 ± 6.4	20.7 ± 6.4	20.8 ± 6.4	20.6 ± 6.1	19.5 ± 6.6	0.383
Acute/worsening chronic HF	100 (24.3%)	22 (21.2%)	22 (21.6%)	29 (28.2%)	27 (26.5%)	0.366
NT-proBNP (pg/mL)	3541 (1952–6909)	4273.0 (2287.5–8362.0)	3573.5 (1337.3–6935.8)	3361.0 (1933.0–6310.0)	2939.5 (1790.0–6616.8)	0.336
NYHA class, III-IV	335 (81.5%)	87 (83.7%)	78 (76.5%)	88 (85.4%)	82 (80.4%)	0.559
**Medical history**						
Coronary artery disease	191 (46.5%)	52 (50.0%)	51 (50.0%)	37 (35.9%)	51 (50.0%)	0.105
Myocardial infarction	87 (21.2%)	29 (27.9%)	23 (22.5%)	13 (12.6%)	22 (21.6%)	0.059
PCI	48 (11.7%)	11 (10.6%)	11 (10.8%)	11 (10.7%)	15 (14.7%)	0.751
CABG	9 (2.2%)	1 (1.0%)	2 (2.0%)	3 (2.9%)	3 (2.9%)	0.734
Dilated cardiomyopathy	53 (12.9%)	16 (15.4%)	15 (14.7%)	13 (12.6%)	9 (8.8%)	0.497
Valvular heart disease	39 (9.5%)	11 (10.6%)	10 (9.8%)	8 (7.8%)	10 (9.8%)	0.914
Congenital heart disease	4 (1.0%)	1 (1.0%)	1 (1.0%)	0 (0.0%)	2 (2.0%)	0.563
Hypertension	168 (40.9%)	36 (34.6%)	43 (42.2%)	39 (37.9%)	50 (49.0%)	0.176
Diabetes mellitus	112 (27.3%)	22 (21.2%)	33 (32.4%)	24 (23.3%)	33 (32.4%)	0.142
CKD	40 (9.7%)	9 (8.7%)	13 (12.7%)	8 (7.8%)	10 (9.8%)	0.649
Prior history of HF	295 (71.8%)	80 (76.9%)	69 (67.6%)	71 (68.9%)	75 (73.5%)	0.426
AF	121 (29.4%)	36 (34.6%)	32 (31.4%)	25 (24.3%)	28 (27.5%)	0.385
**Echocardiography findings**						
IVST (mm)	10.0 (9.0–10.7)	10.0 (9.0–11.0)	10.0 (9.0–10.1)	10.0 (9.0–10.0)	10.0 (9.0–11.0)	0.278
PWT (mm)	10.0 (9.0–10.0)	9.0 (9.0–10.0)	9.0 (9.0–10.0)	9.0 (9.0–10.0)	9.0 (8.0–10.0)	0.592
LVDv (mL)	158.0 (107.0–211.0)	169.0 (107.5–239.0)	161.0 (101.5–206.3)	144.0 (103.0–208.0)	148.5 (113.3–207.3)	0.456
LVSv (mL)	83.0 (44.0–133.0)	101.5 (48.0–159.5)	83.0 (42.0–136.0)	68.0 (39.0–127.0)	86.5 (45.8–132.0)	0.451
LVEF (%)	48.0 (36.0–59.0)	45.5 (35.0–58.0)	46.0 (35.0–59.0)	50.0 (40.0–60.0)	50.5 (35.0–60.0)	0.305
LAD (mm)	41.0 (37.0–47.0)	43.0 (38.0–47.0)	40.0 (36.0–46.3)	39.0 (37.0–45.0)	41.0 (36.0–47.0)	0.047
**Medications**						
Beta-blocker	258 (62.8%)	64 (61.5%)	62 (60.8%)	64 (62.15)	68 (66.7%)	0.821
CCB	94 (22.9%)	17 (16.3%)	31 (30.4%)	18 (17.5%)	28 (27.5%)	0.034
Statins	189 (46.0%)	43 (41.3%)	51 (50.0%)	49 (47.6%)	46 (45.1%)	0.637
ARB	125 (30.4%)	27 (26.0%)	38 (37.3%)	27 (26.2%)	33 (32.4%)	0.234
ACE-I	146 (35.5%)	38 (36.5%)	35 (34.3%)	39 (37.9%)	34 (33.3%)	0.903
Diuretics	247 (60.1%)	62 (59.6%)	66 (64.7%)	59 (57.3%)	60 (58.8%)	0.724

Data are presented as mean (standard deviation), median (interquartile range), or n (%).

*BMI* body mass index, *NT-proBNP* n-terminal pro brain natriuretic peptide, *CKD* chronic kidney disease, *AF* atrial fibrillation, *IVST* interventricular septum thickness, *PWT* posterior wall thickness, *LVDv* left ventricular end-diastolic volume, *LVSv* left ventricular end-systolic volume, *LVEF* left ventricular ejection fraction, *LAD* left atrial dimension, *CCB* calcium-channel blocker, *ARB* angiotensin receptor blocker, *ACE-I* angiotensin converting enzyme inhibitor.

Among medical history, coronary artery disease (45%) was the most common ischemic etiology, followed by dilated cardiomyopathy (14%). Hypertension and diabetes mellitus were identified in 40.9% and 27.3% of patients, respectively. Moreover, 29.4% of patients had concomitant atrial fibrillation. The mean serum level of Se of all included patients with HF was 68.3 ± 27.7 µg/L. When patients were categorized by quartiles of serum Se concentration, there was nonsignificant difference between the four groups (Table 1).

### Comparison between survival and deceased patients

During a median of 23 (15–37) months follow-up, 131 (31.9%) death and 216 (52.6%) rehospitalization for HF were observed. Compared to survival patients with HF, those deceased patients had a reduced serum Se concentration (72.4 ± 25.9 µg/L vs. 59.4 ± 29.4 µg/L; *P* < 0.001) (Table 2). Moreover, those patients were more often older, and more likely to have higher body mass index (BMI) value, higher NT-proBNP level, and larger left atrial diameter. Chronic kidney disease was also more frequently in patients who died, while statin and angiotensin converting enzyme inhibitor were less prescribed to them.

**Table 2:** Comparison of survival and deceased patients with heart failure.

Variables	Survival patients (n = 280)	Deceased patients (n = 131)	*P*
Age (years)	61.2 ± 16.0	65.1 ± 15.4	0.022
Female	118 (42.1%)	65 (49.6%)	0.167
BMI (kg/m^2^)	26.1 ± 4.6	27.9 ± 4.6	< 0.001
Current smoker	58 (20.7%)	24 (18.3%)	0.599
Current drinker	42 (15.1%)	19 (14.5%)	1.000
**Clinical features**			
Prior history of HF	199 (71.1%)	96 (73.3%)	0.643
HF duration (months)	20.3 ± 6.5	20.7 ± 6.2	0.584
Acute/worsening chronic HF	76 (27.1%)	45 (34.4%)	0.135
NT-proBNP (pg/mL)	2819 (1467–5485)	5720 (2813–10,000)	< 0.001
NYHA class, III–IV	227 (81.1%)	108 (82.4%)	0.739
Serum selenium (µg/L)	72.4 ± 25.9	59.4 ± 29.4	< 0.001
**Medical history**			
Coronary artery diseas	124 (44.3%)	67 (51.1%)	0.194
Myocardial infarction	54 (19.3%)	33 (25.2%)	0.172
PCI	27 (9.6%)	21 (16.0%)	0.060
CABG	4 (1.4%)	5 (3.8%)	0.123
Dilated cardiomyopathy	34 (12.1%)	19 (14.5%)	0.506
Valvular heart disease	23 (8.2%)	16 (12.2%)	0.197
Congenital heart disease	1 (0.4%)	3 (2.3%)	0.063
Hypertension	118 (42.1%)	50 (38.2%)	0.454
Diabetes mellitus	73 (26.1%)	39 (29.8%)	0.476
CKD	20 (7.1%)	20 (15.3%)	0.012
Prior history of HF	199 (71.1%)	96 (73.3%)	0.724
AF	76 (62.8%)	45 (37.2%)	0.163
**Echocardiography findings**			
IVST (mm)	10.0 (9.0–10.0)	10.0 (9.0–11.0)	0.291
PWT (mm)	9.0 (9.0–10.0)	9.0 (9.0–10.0)	0.079
LVDv (mL)	154.0 (106.3–204.0)	168.0 (109.0–236.0)	0.130
LVSv (mL)	77.0 (43.0–128.8)	101.0 (47.0–137.0)	0.196
LVEF (%)	48.0 (36.0–60.0)	46.0 (35.0–59.0)	0.604
LAD (mm)	40.0 (36.0–45.0)	43.0 (38.0–50.0)	0.001
**Medications, n (%)**			
Beta-blocker	184 (65.7%)	74 (56.5%)	0.080
CCB	67 (23.9%)	27 (20.6%)	0.529
Statins	145 (51.8%)	44 (33.6%)	0.001
ARB	88 (31.4%)	37 (28.2%)	0.566
ACE-I	109 (38.9%)	37 (28.2%)	0.036
Diuretics	167 (59.6%)	80 (61.1%)	0.783

Data are presented as mean (standard deviation), median (interquartile range), or n (%). Abbreviation as in Table 1.

### Serum Se concentration and all-cause mortality

**Figure 1:**
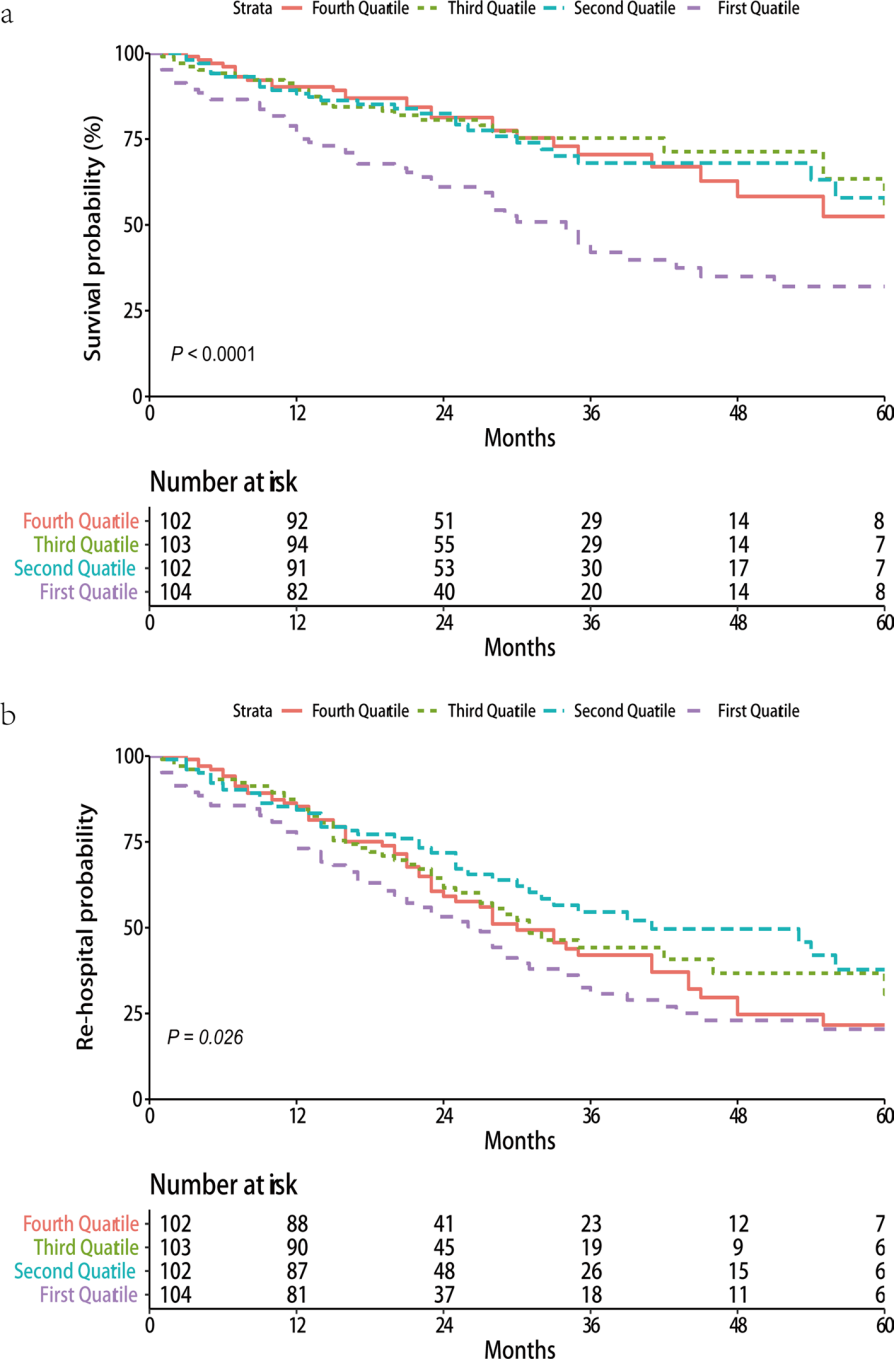
Kaplan–Meier survival curve for all-cause mortality (**a**) and rehospitalization (**b**) among patients with heart failure.

**Figure 2:**
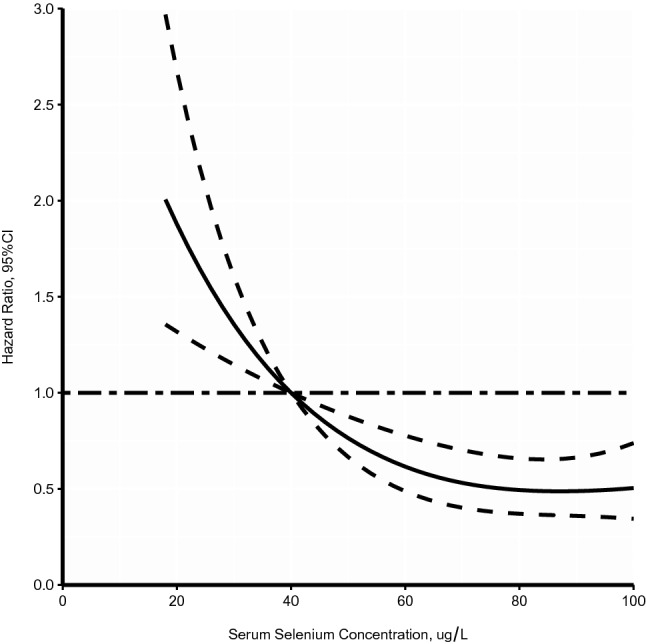
Shapes of the concentration–response relationships of serum selenium and risk of mortality.

There were 26 (25.5%) all-cause mortality events occurred in patients with HF in the 1st quartile serum Se concentration, 26 (25.2%) events in the 2nd quartile, 27 (26.5%) events in the 3rd quartile, and 52 (50.0%) events in the 4th quartile, respectively. The Kaplan–Meier survival examination showed the poorest prognosis in patients with HF with the lowest serum Se concentration (log-rank *P *= 0.005) when compared with the remained three groups (Fig. 1). In comparison with patients in the highest quartile, those with the lowest quartile (17.4–44.35 µg/L) were associated with increased risk of all-cause mortality [adjusted hazards ratios (95% CI) 2.32 (1.43–3.77); *P*_trend_ = 0.001] (Table 3; Fig. 2). In subgroup analyses, the simialr effect directions with the main resutls were obeserved in each subgroup although the magnitudes were attenuated (Tables 4, 5). And nonsignificant interactions existed in different subgroups.

**Table 3:** The associations of serum selenium with prognosis in patients with heart failure.

	Model 1		Model 2		Model 3	
	HR (95% CI)	*P*	HR (95% CI)	*P*	HR (95% CI)	*P*
**Rehospitalization**						
4th quartile (94.15–116.7 µg/L)	Ref		Ref		Ref	
3rd quartile (68.05–94.15 µg/L)	0.92 (0.63–1.34)	0.648	0.99 (0.67–1.46)	0.940	0.99 (0.67–1.47)	0.953
2nd quartile (44.35–68.05 µg/L)	0.72 (0.48–1.07)	0.103	0.77 (0.51–1.15)	0.201	0.76 (0.51–1.15)	0.764
1st quartile (17.4–44.35 µg/L)	1.34 (0.94–1.92)	0.105	1.30 (0.90–1.88)	0.160	1.31 (0.90–1.89)	0.155
Trend test		0.247		0.330		0.324
Every 5 µg/L decrease	1.08 (0.96–1.01)	0.132	1.02 (0.99–1.04)	0.182	1.02 (0.99–1.04)	0.185
**All-cause mortality**						
4th quartile (94.15–116.7 µg/L)	Ref		Ref		Ref	
3rd quartile (68.05–94.15 µg/L)	1.00 (0.58–1.72)	0.990	1.15 (0.66–2.00)	0.628	1.12 (0.64–1.96)	0.681
2nd quartile (44.35–68.05 µg/L)	1.02 (0.59–1.75)	0.948	1.06 (0.62–1.84)	0.824	1.06 (0.61–1.84)	0.831
1st quartile (17.4–44.35 µg/L)	2.40 (1.50–3.85)	< 0.001	2.34 (1.44–3.80)	0.001	2.32 (1.43–3.77)	0.001
Trend test		< 0.001		0.001		0.001
Every 5 µg/L decrease	1.08 (1.04–1.12)	< 0.001	1.07 (1.04–1.11)	< 0.001	1.07 (1.04–1.11)	< 0.001

Data are completed for all variables. Model 1: no adjustment; Model 2: adjusted for age, sex, body mass index, smoking, drinking, hypertension, diabetes, chronic kidney disease, prior history of heart failure, and atrial fibrillation; Model 3: further adjusted for medication.

**Table 4:** Subgroup analyses for associations between serum selenium and the risk of mortality in patients with heart failure.

Covariates	Death/participants	HR (95% CI)	*P*
**Sex**			0.862
Male	66/228	1.07 (1.02–1.12)	0.007
Female	65/183	1.07 (1.02–1.13)	0.004
**Age (years)**			0.894
< 75	95/309	1.06 (1.02–1.10)	0.002
≥ 75	36/102	1.11 (1.03–1.20)	0.009
**BMI (kg/m**^**2**^**)**			0.105
< 25	46/170	1.02 (0.96–1.08)	0.581
≥ 25	85/241	1.09 (1.05–1.14)	< 0.001
**Smoking status**			0.439
Never	107/329	1.06 (1.02–1.15)	0.001
Ever	24/82	1.13 (1.03–1.25)	0.011
**Alcohol drinking**			0.273
Seldom	112/350	1.06 (1.02–1.10)	< 0.001
Occasional or regular	19/61	1.15 (1.03–1.29)	0.016

Adjusted for age, sex (except in the sex-stratified analysis), body mass index, smoking (except in the smoking-stratified analysis), drinking (except in the alcohol-stratified analysis), hypertension, diabetes, chronic kidney disease, prior history of heart failure, atrial fibrillation and medication.

**Table 5:** Subgroup analyses for associations between serum selenium and the risk of rehospitalization in patients with heart failure.

Covariates	Rehospitalization/participants	HR (95% CI)	P*
**Sex**			0.395
Male	66/228	1.03 (1.00–1.07)	0.097
Female	65/183	1.00 (0.97–1.04)	0.882
**Age (years)**			0.882
< 75	95/309	1.01 (0.97–1.04)	0.369
≥ 75	36/102	1.03 (0.98- 1.08)	0.276
**BMI (kg/m**^**2**^**)**			0.012
< 25	46/170	0.96 (0.92–1.01)	0.090
≥ 25	85/241	1.04 (1.01–1.08)	0.009
**Smoking status**			0.120
No	107/329	1.01 (0.98–1.04)	0.704
Yes	24/82	1.06 (1.00–1.12)	0.061
**Alcohol drinking**			0.092
No	112/350	1.01 (0.98–1.04)	0.598
Yes	19/61	1.08 (0.99–1.16)	0.071

*Adjusted for age, sex (except in the sex-stratified analysis), body mass index, smoking (except in the smoking-stratified analysis), drinking (except in the alcohol-stratified analysis), hypertension, diabetes, chronic kidney disease, prior history of heart failure, atrial fibrillation and medication.

### Serum Se concentration and rehospitalization

There were 57 (55.9%) rehospitalization for HF events occurred in patients with HF in the 1st quartile serum Se concentration, 50 (48.5%) events in the 2nd quartile , 43 (42.2%) events in the 3rd quartile, and 66 (63.5%) events in the 4th quartile. The Kaplan–Meier survival curve revealed the similar incidences of rehospitalization within the four groups patients with HF, even though the difference was significant (log-rank P = 0.026) (Fig. 1). The COX survival models failed to find a positive association between serum Se concentration with rehospitalization event in patients with HF. Subgroup analyses demenstrated the simialr findings with the main resutls across different subgroups.

## Discussion

To date, we firstly investigated the relationship between serum Se concentration and prognosis in Chinese patients with HF. Our findings suggest that serum Se concentration was decreased in those patients died during follow-up, and the lower serum Se level was significantly associated with all-cause mortality in patients with HF. However, similar association was not identified between this marker and rehospitalization for HF in these patients.

The Se intake in human body has been known to largely depend on the level of Se in soil, thus leading to a variety of serum Se concentration among different population. The Se deficiency was the well-defined cause of an endemic cardiomyopathy called Keshan disease in China, which was characterized as congestive HF and sequent high case-fatality rate^11^. With the increased knowledge in metabolism of trace elements, the role of Se has been related to morbidity and mortality in several diseases. In a Germany study included 1731 individuals with coronary artery disease, low Se concentration was found to be associated with future cardiovascular death in patients with acute coronary syndrome during a median follow-up of 6.1 years^15^. Lee et al. had reported that low serum selenium levels in patients with respiratory diseases have a significant relationship with poor prognosis on admission^16^. However, the randomized controlled trials with Se supplements failed to find preventive effect of Se on the incidence of type 2 diabetes, cancers, and thyroid disease^17–19^. These results have caused more confusions about the real physiological role of Se.

Despite all this, there are still raising interests upon the potential association between serum Se level and the outcomes of HF^20^. The important consideration is the possible benefit from Se homeostasis maintenance to improve the high prevalence of malnutrition and poor prognosis in patients with HF^21^. Although the decreased Se level was frequent in patients with HF compared to health controls^12,22,23^, the definitely correlation between this reduction and long-term survival was not well-established. Recently, an European multinational prospective cohort study revealed a 20.4% of Se deficiency among 2516 worsening HF patients, and found this deficiency in HF patients is independently associated with impaired exercise tolerance and a 50% higher mortality rate^13^. In the current study, we also observed a high prevalence of Se deprivation in Chinese patients with HF in the setting of previously reported reference of 70 µg/L^13^. Furthermore, our results showed the trend of inverse association between serum Se level significantly and all-cause mortality in patients with HF, which was in line with prior study’s findings. As a result, these aforementioned results have made the foundation for the further randomized controlled trials to assess the feasibility, safety and effectiveness of Se supplements in patients with HF.

The underlying mechanisms of Se in the onset and progression of HF are multifactorial. The Se plays a vital role in the function of the antioxidant GPx enzymes, the main intracellular antioxidant. The GPx enzymes could remove hydrogen peroxide and the harmful lipid hydroperoxides generated in vivo by oxygen-derived species, which are very important defense mechanisms in humans^24^. Another important selenoprotein, called Selenoprotein P, has been identified to be a major extracellular antioxidant, and involved in acute HF^25^. Se is also known to affect thyroid hormone metabolism and the synthesis and activity of deiodinases, enzymes converting thyroxin into the biologically active riiodothyronine^26^. In animal experiments, Se deficiency led to reactive myocardial fibrosis and systolic dysfunction accompanied by increased myocardial oxidant stress via effects on Redox–Methylation balance^27^. Furthermore, Se supplementation lowers insulin resistance and markers of cardio-metabolic risk in patients with congestive HF, which might inform the potential benefits of Se supplements^28^.

There are some limitations need interpreting. First, this study was limited by retrospective design, and the sample size might be unable to reach statistical power especially in subgroup analyses. Second, we could not identify a “true” Se deficiency status because of the missing of reference ranges for serum Se level in China. As such, reference ranges from European countries might not be suggested to apply in Chinese population considering China was classified as low Se region. Third, even though a number of confounders were adjusted, we cannot rule out the possibility that unmeasured factors or residual confounders might contribute to the associations our study observed. Finally, the observational data analysis of the associations between markers of Se status and prognosis in patients with HF make it impossible for us to investigate the causal conclusion. However, the prospective association from European cohort also suggests that lower serum Se levels were positively associated with long-term prognosis in patients with HF.

In conclusion, serum low Se concentration was common in Chinese patients with HF. The lower serum Se level was significantly associated with increased risk of all-cause mortality in these patients.

## Methods

### Patients

Consecutive patients with acute or chronic HF aged 18 years or older were consecutively included for initial screening. All patients were from the Nanyang Central Hospital, Nanyang, Henan, China, where was defined as adequate- and low-selenium area. The electronic medical records were retrospectively reviewed and the diagnosis of HF was based on typical clinical manifestations and signs, such as breathlessness, paroxysmal nocturnal dyspnea, reduced exercise tolerance, jugular vein distension or peripheral edema, plasma NT-proBNP and echocardiography findings according to the current guideline^29^. A cutoff value of 125 pg/mL or 300 pg/mL for NT-proBNP was used in the diagnostic algorithm of acute HF and chronic HF, respectively. The patients with serum Se examination would be finally included after the following exclusion criteria were all satisfied: thyroid disease, treatment with amiodarone or glucocorticoids, and severe systemic disease such as infectious, inflammatory, autoimmune, or neoplastic disease. At admission, patients with HF received the standard treatments that guideline recommended, whereas the etiological treatments were dependent on patient's condition and at physician’s discretion. The study protocol was in accordance with the Declaration of Helsinki and was approved by by the Ethics Committee of Nanyang Central Hospital. The informed consent was obtained from all participants before participating the study.

### Data collection

Information on demography, laboratory tests, co-morbidities, echocardiography examinations and medication treatment were collected at patient’s first admission. After admission to hospital, five milliliter blood sample was withdrawn from each participants after a 12 h fasting period. Serum was immediately separated from whole blood and stored at − 80 ℃ until analysis. The carbon-furnace atomic-absorption spectrometry and Zeeman compensation was used to determine the serum Se concentrations by a Spectra AA 220 Z (Varian). NT-proBNP levels were measured using electrochemiluminescent immunoassay (ECLIA) method by a VIDAS 30 (bioMerieux). The transthoracic echocardiographic evaluation was performed during hospitalization and records were reviewed by a senior echocardiographer.

### Outcomes and follow-up

The primary outcome of interest in this study was all-cause mortality. The secondary outcome was rehospitalization for HF. All patients were followed up via clinic visit, phone or internet interview, until death or rehospitalization event occurred, or up to June 2020 or the last follow-up if index event did not occur. The survival data was censored at the end of the follow-up time.

### Statistical methods

The numerical variables are presented as mean (standard deviations [SD]) or median (interquartile range [IQR]) according to distribution patterns. The categorical variables are presented as count with frequencies. One-way analysis of variance, the Kruskal–Wallis test, the Student’s t test and the Pearson’s chi-square test were applied, as indicated, for comparisons of between-group difference. The study participants were divided into quartiles according to their serum Se concentrations. Kaplan–Meier survival curves were constructed and the log-rank test was used to compare either death or rehospitalizations as events in different groups. The Cox proportional hazard models were adopted to estimate the association of serum Se levels with the primary outcome and secondary outcome. Three models were adopted: Model 1, without any adjustment; Model 2, adjusting for age, sex, BMI, NT-proBNP, smoking, drinking, hypertension, diabetes, chronic kidney disease, prior history of HF, and atrial fibrillation; Model 3, further adjusting for medication treatment. The concentration–response curve was then evaluated using a natural cubic spline.

Subgroup analyses were also conducted to investigate whether the associations were modified by sex (male or female), age (< 75 or ≥ 75 years), BMI (< 25 or ≥ 25 kg/m^2^), smoking status (never or ever), and drinking (yes or no). Each potential modifier was examined in a separate model by adding a multiplicative interaction term (i.e., potential modifier ∗ Se concentration).

All statistical analyses were conducted using SPSS 21.0 for Windows (SPSS, Inc., Chicago, USA), and two-sided *P* < 0.05 was considered statistically significant.

### Ethical approval

The study protocol was in accordance with the Declaration of Helsinki and was approved by by the Ethics Committee of Nanyang Central Hospital. The informed consent was obtained from all participants before participating the study.

## Data Availability

Additional data are available from the corresponding author for reasonable requesting.
